# Comprehensive Immunisation Among Children Aged 12–23 Months in India—A Multilevel Modelling Analysis From the National Family Health Survey (2015–21)

**DOI:** 10.1002/puh2.70329

**Published:** 2026-08-11

**Authors:** Mansi Malik, Saurav Basu, Siaa Girotra, Satyajit Kundu

**Affiliations:** ^1^ Indian Institute of Public Health—Delhi Public Health Foundation of India Delhi India; ^2^ Department of Community Medicine ESIC Medical College and Hospital Kolkata India; ^3^ ARCED Foundation Dhaka Bangladesh; ^4^ Public Health, School of Medicine and Dentistry Griffith University Gold Coast Queensland Australia

**Keywords:** immunisation, National Family Health Survey (NFHS), vaccination

## Abstract

Under‐5 child mortality due to vaccine preventable diseases (VPDs) contributes to an estimated burden of ∼5.2 million deaths globally, mostly within low‐ and middle‐income countries (LMICs). Despite ongoing efforts to expand immunisation coverage, gaps in immunisation remain a persistent public health concern, underscoring the need to examine recent trends and determinants of full immunisation in India. The objective of this study is to estimate vaccination coverage and identify factors associated with full immunisation among children aged 12–23 months from 2015 to 2021. We utilised the National Family Health Survey‐4 (NFHS‐4) and NFHS‐5 datasets to determine the vaccination status (fully vaccinated/not fully vaccinated) among children aged 12–23 months using multilevel logistic regression analysis. The full vaccination coverage increased from 62.54% [95% CI: 61.81, 63.26] in 2015–16 (NFHS‐4) to 76.81% [95% CI: 76.18, 77.42] in 2019–21 (NFHS‐5). The factors associated with significantly higher odds of full vaccination in both NFHS‐4 and NFHS‐5 included higher maternal age and education, higher wealth quintile, having ≥4 antenatal care visits, institutional delivery, pregnancy registration and exposure to media. The lower odds of full immunisation were associated with having more than one child and being Muslim in both NFHS‐4 and NFHS‐5. The elimination of rural residence as a significant factor for lower child vaccination odds in NFHS‐5, a contrast to NFHS‐4, suggests that national health policies have become more effective at reducing geographical disparities and therefore more equitable. With increasing saturation of vaccination coverage, the government programme needs a specific focus on addressing key determinants associated with vaccine uptake to achieve complete vaccination.

## Abstract 2

Full immunisation coverage rose from 62.5% in 2015–16 to 76.8% in 2019–21. Higher maternal age, education, wealth, antenatal care, institutional delivery, pregnancy registration and media exposure improved vaccination odds, while having multiple children and Muslim religious affiliation were linked to lower coverage in both NFHS rounds.

## 1 Introduction

Childhood vaccinations globally prevent approximately 4 million deaths annually, making them one of the most effective public health interventions ever deployed. In 2019, the global toll of under‐5 child mortality, predominantly due to avoidable factors like the absence of childhood vaccination, reached 5.2 million, with India and Nigeria constituting one‐fifth of this burden [1].

In India, infectious diseases contribute significantly to childhood mortality and morbidity, wherein nearly one million children die before their fifth birthday [2] despite the initiation of the landmark Universal Immunisation Programme (UIP) in 1984. In the following three decades, newer vaccines, including pentavalent, tetanus and adult diphtheria (TD), inactivated poliovirus vaccine (IPV), measles–rubella vaccine (MR), rotavirus vaccine and pneumococcal conjugate vaccine (PCV), have been incorporated within the national immunisation schedule (NIS) of the UIP [3, 4, 5, 6].

Despite the availability of vaccination services free of cost in the Indian public health sector, a high burden of children remains unvaccinated or partially vaccinated due to deficiencies in vaccine accessibility and hesitancy [7, 8, 9]. Several factors that contribute to low vaccination coverage include suboptimal quality of functioning and accessibility of healthcare facilities; challenges in reaching rural, remote, tribal and migrant populations; populations lacking awareness or misinformation; and vaccine hesitancy due to irrational fear of vaccine‐related side effects [10].

The Government of India launched the Mission Indradhanush programme in 2014 and Intensified Mission Indradhanush (IMI; Kawach) in 2017 to expedite efforts towards universal immunisation, especially in remote and underserved areas [11]. The target of MI was achieving 90% full immunisation coverage in the country by 2020, with emphasis on high‐focus districts having the highest burden of unvaccinated children.

Comprehensive childhood immunisation is a cornerstone of public health, crucial for reducing child morbidity and mortality. Although prior studies have explored immunisation coverage in India, many suffer from significant methodological limitations. A substantial number of these studies are based on small sample sizes and a single‐site focus, which severely restricts the generalisability of their findings to the diverse landscape of India. Among nationally representative surveys, the NSSO 75th (2017–18) round estimated 59.2% vaccination coverage in children aged 0–5 years, but it coincides with the early‐phase implementation of MI Kawach [12]. The IMI coverage evaluation survey by the Government of India reported an 18.5 percentage point increase in full immunisation coverage compared to previous data from 190 surveyed districts [13].

This study aims to address this critical gap by employing multilevel modelling with data from the National Family Health Survey (NFHS) 2015–21. This approach offers specific advantages that provide a more robust and nuanced understanding of immunisation coverage. Multilevel models explicitly account for the clustering of data, thereby producing more accurate standard errors and reliable estimates. Most importantly, this method allows for the simultaneous investigation of both individual‐level determinants and higher level contextual community‐related factors, enabling the identification of insights relevant for health policy.

Although previous studies, such as Saikia et al. [14], Johri et al. [15] and Krishnamoorthy and Rehman [16], have examined determinants of immunisation using NFHS‐5 data, these analyses are limited to single cross‐sectional assessments. Our study contributes to this literature by employing a repeated cross‐sectional multilevel framework using both NFHS‐4 and NFHS‐5 data, enabling the identification of evolving determinants and persistent inequities in immunisation coverage. This approach moves beyond a static snapshot, allowing us to identify persistent multilevel predictors that have remained significant barriers to full immunisation over time; a critical insight for policy that a single cross‐section cannot provide. Furthermore, we conduct stratified multilevel analyses by geography (rural/urban) and socioeconomic profile, which is essential for identifying heterogeneous effects and understanding how the determinants of immunisation coverage differ across these key subpopulations, rather than treating them as mere control variables in an aggregated model.

We therefore conducted this study with the objective of estimating the vaccination coverage, trends and factors associated with full immunisation among the children aged 12–23 months from 2015 to 2021 in India.

## 2 Methods

### 2.1 Data Source and Study Design

Data from the fourth and fifth rounds of the NFHS‐4 and NFHS‐5 in India were used. The NFHS‐4 and NFHS‐5 surveys in India collected data on population and health indicators at district, state/union territory and national levels, with separate estimates for urban and rural areas. Both surveys employed a stratified two‐stage sampling method, utilising villages and Census Enumeration Blocks (CEBs) as primary sampling units (PSUs) in rural and urban areas, respectively. PSUs were selected on the basis of probability proportional to size (PPS), considering factors such as literacy rates and demographics. Household mapping and listing operations preceded the main surveys, with systematic sampling used to select households. NFHS‐4 and NFHS‐5 aimed to ensure representative and reliable data collection across the country, with NFHS‐5 incorporating newer rounds and an expanded scope of indicators. Detailed sampling design and survey protocols are outlined in the respective national reports [17, 18]. The final analytical sample size of children aged 12–23 months from the NFHS‐4 dataset was 48,749, and from NFHS‐5 was 43,730.

### 2.2 Outcome Measure

Vaccination status among children aged 12–23 months was assessed in this study. Children were classified as fully immunised if they had received three doses of the diphtheria–tetanus–pertussis (DTP3)‐containing vaccine (mostly pentavalent), three doses of polio vaccines (excluding zero dose), along with one dose each of the bacillus Calmette–Guérin (BCG) and measles vaccines at any time before the survey. Partially vaccinated children were those who reported any missed doses of the above vaccination, whereas those who did not receive any of the vaccine doses were categorised as not vaccinated/zero‐dose population [19, 20].

Vaccination coverage information was assessed from the vaccination card or from the mother's verbal report. For final analysis, vaccination status was dichotomised as ‘fully vaccinated’ and ‘not fully vaccinated’ (merging partially vaccinated and not vaccinated categories). The rotavirus vaccine was not included in the analysis as it was administered in a few states of India at the time of data collection. Data on the PCV were not included in the current study as information on these was not available in the source datasets.

**FIGURE 1:**
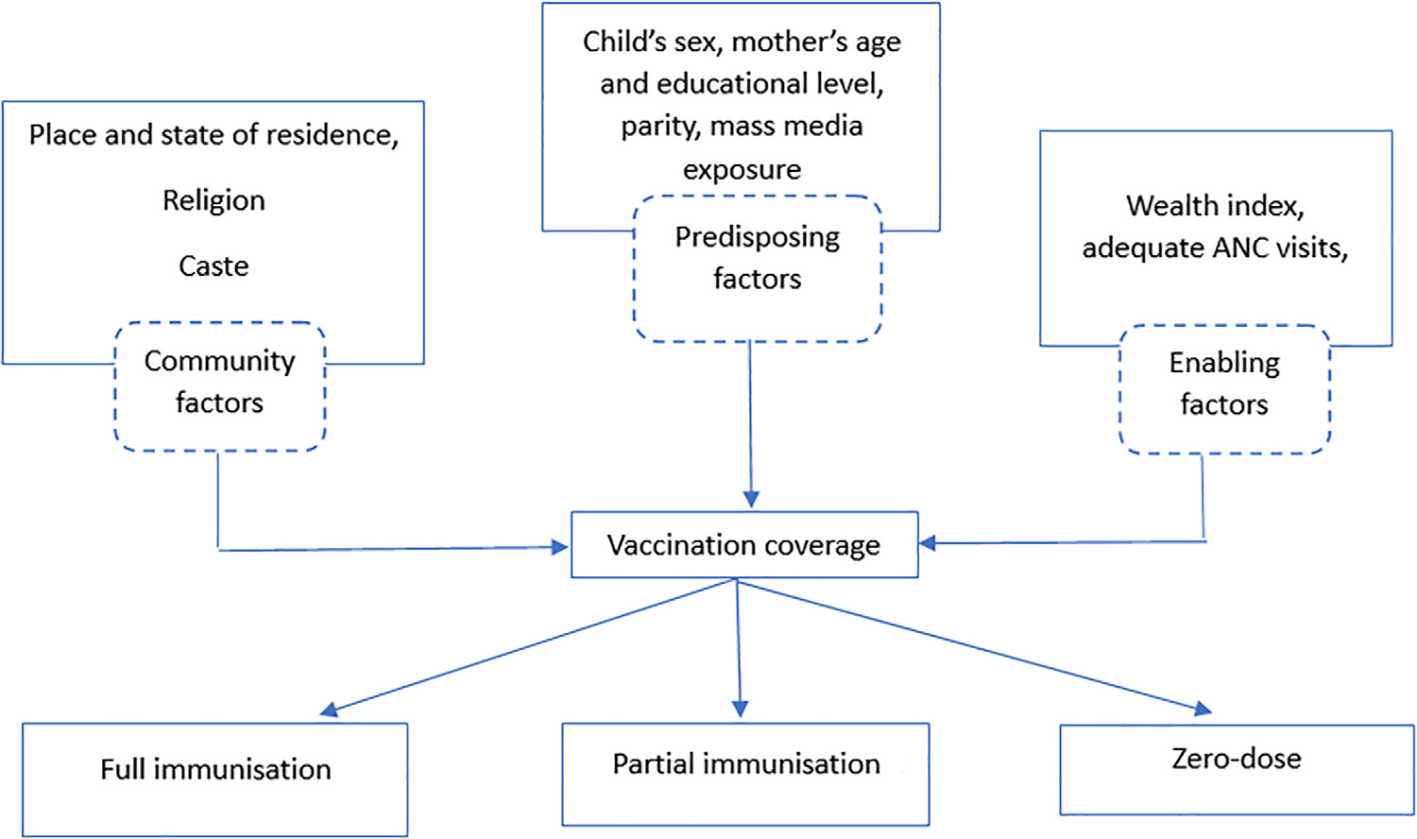
PRECEDE/PROCEED framework for immunisation coverage among children aged 12–23 months in NFHS. ANC, antenatal care.

The PRECEDE/PROCEED framework was used as a conceptual model to identify factors influencing immunisation rates [20, 21]. Our focus was on discerning predisposing, reinforcing and enabling factors linked to achieving full immunisation status among children (Figure 1).

In the context of childhood vaccination, we operationalised the framework's components as follows:

- Predisposing factors: These refer to intrinsic caregiver characteristics that shape their motivation to immunise, such as their knowledge (e.g., of disease severity and vaccine schedules), attitudes (e.g., vaccine efficacy) and beliefs (e.g., vaccine safety). As the NFHS does not capture these specific knowledge, attitude and practice (KAP) variables, we selected established proxy variables known to correlate with these constructs. Our analysis therefore included maternal age, educational level, exposure to mass media (as proxies for health literacy and autonomy), maternal parity (shapes health beliefs and perceptions) and the number of antenatal care (ANC) visits (as a proxy for engagement with the health system and exposure to health information).
- Enabling factors: These are the external, structural or systemic conditions that facilitate or hinder access to vaccination services, including their availability, accessibility and affordability. To measure these, our analysis included the place of delivery (as a proxy for accessibility to vaccination at birth), wealth index (as a proxy for affordability and the ability to overcome indirect costs), pregnancy registration (formal entry point into the maternal and child health system), the adequacy of ANC visits (as a measure of successful access to and utilisation of routine health services) and the country region representative of broad external factors that influence health systems and hinder or facilitate access.
- Reinforcing factors: These factors include the social feedback, support and community norms that encourage or discourage the vaccination behaviour. We operationalised this concept using community‐level variables that serve as proxies for shared social norms and support systems. These variables included religion, caste and place of residence (rural/urban).

To align this theoretical framework with the hierarchical structure of the NFHS data, a multilevel logistic regression (MLLR) model was employed. The individual‐level variables (Level 1) combined the predisposing and enabling factors, such as maternal age, education, wealth quintile and ANC visits, whereas the community‐level factors (Level 2), including place of residence, religion and caste, were used to account for the clustering of data.

### 2.3 Independent Variables

Individual‐level explanatory variables included mother's age, mother's education, ANC visit, place of residence, division, gender, place of delivery and number of children. Maternal age was categorised as ‘less than 25 years’, ‘25–34 years’ and ‘above 34 years’. Maternal education was classified into ‘no education’, ‘primary’, ‘secondary’ and ‘higher’ education.

Mothers were classified as having mass media exposure if they reported reading newspapers or magazines, listening to the radio or watching television. Place of delivery was categorised as ‘home’ or ‘Institutional’ (public and private health care facilities). ANC visits of mothers were categorised according to the recommended minimum threshold of more than four visits by the WHO as <4 and ≥4 ANC visits.

Community‐level factors consisted of the place of residence (urban or rural); religion (Hindu/Muslim/Others); and caste (General, Scheduled Caste [SC], Scheduled Tribe [ST], Other Backward class [OBC]) [22].

### 2.4 Statistical Analysis

In this study, we performed repeated cross‐sectional analyses using data from the NFHS‐4 and NFHS‐5 to understand the overall vaccination coverage as well as the changes in vaccination coverage over the years. We used the ‘svy’ command for assigning the sample weight to adjust for the clustering effect and sample stratification.

We described the weighted prevalence of fully vaccinated children across different states. Considering the two‐stage stratified cluster sampling of NFHS datasets, we used MLLR analysis to identify the factors influencing full vaccination coverage by reducing the cluster effects. Level‐1 factors included individual‐level variables (e.g., maternal education and ANC visits), whereas Level‐2 included community‐level variables (e.g., place of residence and religion) that captured clustering effects. This is because a single‐level analysis would not be appropriate for analysing such data sets that have hierarchical structures. We considered the enumeration units (clusters) as Level‐2 factor for all the regression models.

Multicollinearity among independent variables was checked using the variance inflation factor (VIF). To understand the variation of full immunisation coverage among different enumeration units, the intra‐cluster correlation (ICC) was estimated. We further performed stratified analysis on the basis of place of residence and socio‐economic status. The adjusted odds ratios (AORs) along with 95% confidence intervals (CIs) were used to interpret the findings, and 5% significance level was considered. All analyses were performed using STATA v.15.1.

### 2.5 Ethical Considerations

This study is a secondary data analysis of de‐identified data from the publicly accessible NFHS datasets from Rounds 4 and 5. The original survey participants voluntarily provided signed informed consent, and the survey protocol received approval from the Institutional Review Board at the International Institute for Population Sciences (IIPS), Mumbai. After reviewing the submitted proposal by the authors, the Demographic Health Survey (DHS) granted access to the dataset.

## 3 Results

The full vaccination coverage among children aged 12–23 months in India increased from 62.54% [95% CI: 61.81, 63.26] in 2015–16 (NFHS‐4) to 76.81% [95% CI: 76.18, 77.42] in 2019–21 (NFHS‐5). Furthermore, the proportion of zero‐dose children has decreased from 5.96% [95% CI: 5.62, 6.33] in NFHS‐4 to 3.52% [95% CI: 3.23, 3.83] in NFHS‐5. These findings also reflect the increased coverage of DPT‐containing vaccine or pentavalent (NFHS‐4: 66.42%; NFHS‐5: 68.04%) and BCG (NFHS‐4: 92.04%; NFHS‐5: 95.32%) (Table 1). Similarly, there is an increase in the uptake of oral polio vaccine (OPV) doses (0–3), whereas the coverage for measles (first dose) has increased from 81.63% in 2015–16 to 88.54% in 2019–21. As the rotavirus vaccine and fractional‐dose inactivated poliovirus (fIPV) vaccine were introduced in 2016, data for these vaccines were only available in NFHS‐5. Coverage of rotavirus vaccine (1–3) was estimated to be ∼40% while that of fIPV (1–2) was ∼70% in India (Table 1).

**TABLE 1:** Vaccination coverage weighted percentages in the 12–23 age group.

	NFHS‐4	NFHS‐5
Vaccine type	% (95% CI)	% (95% CI)
**Hepatitis B at birth**	66.42 (65.81, 67.02)	68.04 (67.41, 68.70)
**BCG**	92.05 (91.70, 92.39)	95.32 (95.02, 95.61)
**OPV**		
OPV 0	79.25 (78.74, 79.74)	85.63 (85.16, 86.08)
OPV 1	90.59 (90.22, 90.96)	92.00 (91.60, 92.30)
OPV 2	86.10 (85.66, 86.54)	88.50 (88.10, 88.93)
OPV 3	73.69 (73.11, 74.26)	81.46 (80.92, 82.00)
**DPT**		
1st dose	89.68 (89.28, 90.07)	93.40 (93.10,93.73)
2nd dose	86.08 (85.64, 86.51)	91.40 (90.98, 91.80)
3rd dose	79.20 (78.69, 79.70)	87.69 (87.23, 88.14)
**Measles (1st dose)**	81.63 (81.14, 82.11)	88.54 (88.09, 88.98)
**Measles (2nd dose)**	NA	31.95 (31.29, 32.62)
**Rotavirus**		
1st dose	NA	43.84 (43.21, 44.48)
2nd dose	NA	41.13 (40.51, 41.77)
3rd dose	NA	37.96 (37.35, 38.58)
**fIPV**	NA	
1st dose	NA	75.51 (74.93, 76.10)
2nd dose	NA	63.47 (62.83, 64.11)
**Full immunisation coverage** ^a^	62.54 (61.81, 63.26)	76.81 (76.18, 77.42)

*Note:* *n*, number who availed vaccine; *N*, total sample.

Abbreviations: BCG, Bacillus Calmette–Guérin; CI, confidence interval; DPT, diphtheria–pertussis–tetanus; fIPV, fractional‐dose inactivated poliovirus vaccine; NA: data not available; NFHS, National Family Health Survey; OPV, oral polio vaccine.

^a^ Children were classified as fully immunised if they had received three doses of the diphtheria–tetanus–pertussis (DTP3)‐containing vaccine (mostly pentavalent), three doses of polio vaccines (excluding zero dose), along with one dose each of the bacillus Calmette–Guérin (BCG) and measles vaccines at any time before the survey.

The overall BCG coverage in India in 2015–16 was 92.02%, which improved to 95.33% in 2019–21 (Figure S1). For the Hepatitis B vaccine at birth (Figure S2), in 2015–16 the overall coverage was 66.42%, which improved marginally to 68.04 in 2019–21. The overall coverage of the measles vaccine (Figure S3) remained consistent at around 81.6% in 2015–16, which improved slightly to 88.5% in 2019–21. The coverage of OPV vaccine (0–3 doses) increased from 62.15% in 2015–16 to 72.63% in 2019–21 (Figure S4). The coverage of the DPT‐containing vaccine (Figure S5) increased from 78.94% to 87.05% in 2019–21.

**FIGURE 2:**
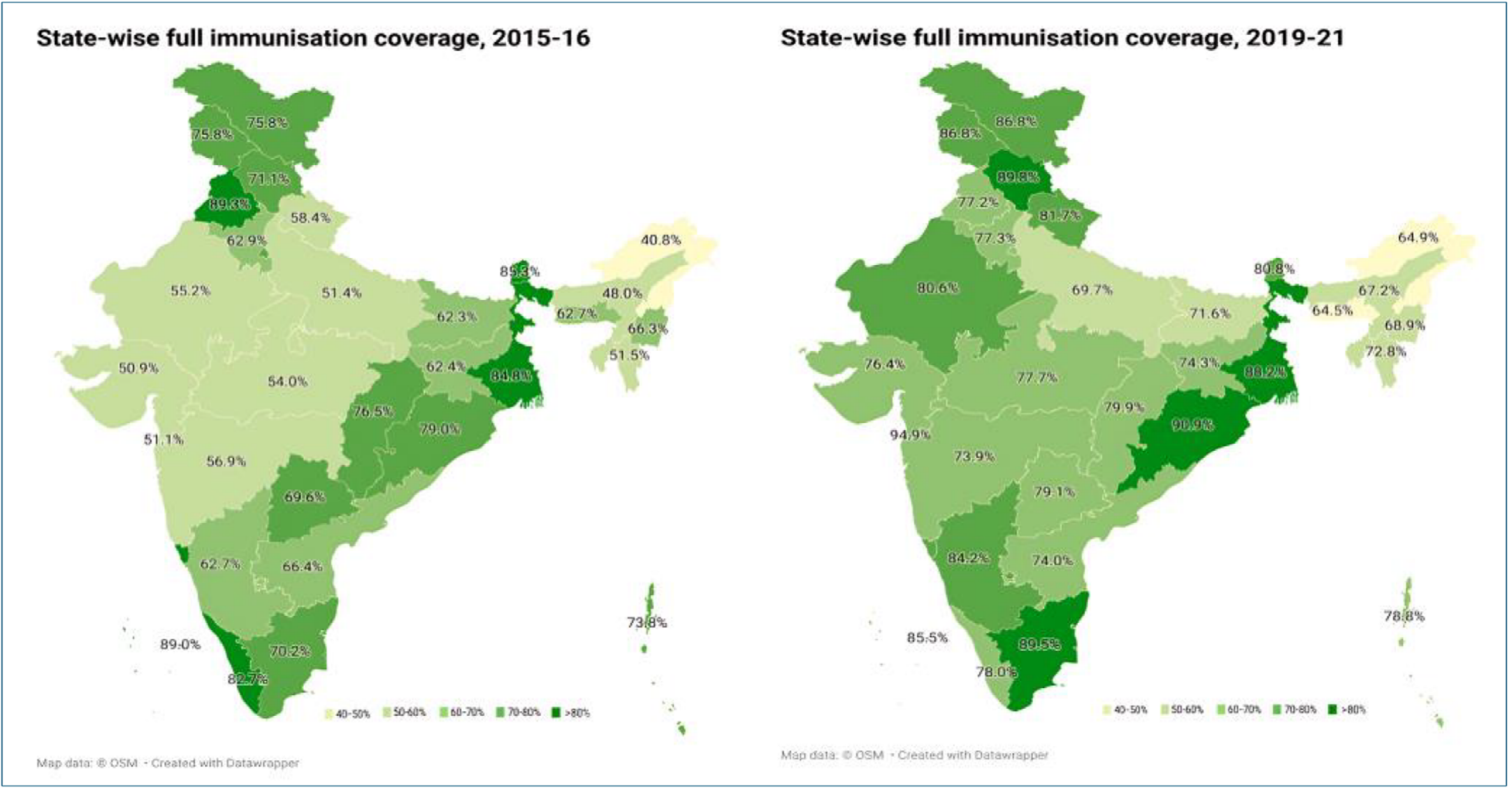
State‐wise immunisation coverage in NFHS‐4 (2015–16) and NFHS‐5 (2019–21).

A major decline in immunisation coverage has been observed in the states of Punjab (−13.5%), Puducherry (−11.1%), Goa (−6.7%), Kerala (−5.7%), Sikkim (−5.2%) and Lakshadweep (−3.9%). On the contrary, union territories and states, such as Dadar and Nagar Haveli and Daman and Diu (+86.0%), Nagaland (+62.0%), Arunachal Pradesh (+59.0%), Gujarat (+50.0%), Rajasthan (+46.0%), Madhya Pradesh (+44.0%) and Mizoram (+41.0%) along with 22 other states observed an increase in the full immunisation coverage (Figure S6). The full vaccine coverage increased from 62.54% in 2015–16 to 76.81% in 2019–21 in India (Table 1). The state‐wise full immunisation coverage over time is shown in Figure 2. Age‐ and sex‐specific full vaccination coverage in NFHS‐5 and NFHS‐4 is represented in Figures S7 and S8, respectively.

On bivariate analysis, higher maternal educational levels, the richest wealth quintile, ≥4 ANC visits, institutional delivery, exposure to mass media and a priori pregnancy registration were significantly associated with full immunisation among children aged 12–23 months in both datasets. On the contrary, increasing maternal age and those having more than one child had lower odds of receiving full immunisation (Table S1).

On adjusted analysis, higher maternal educational levels were associated with higher odds of receiving full vaccination in NFHS‐4 (AOR: 1.42, 95% CI: 1.28, 1.58) and in NFHS‐5 (AOR: 1.18, 95% CI: 1.05, 1.31) compared to mothers who received no formal education (Table 2). Results of adjusted analysis revealed similar findings where children from Muslim families had lower odds of full immunisation in both 2015–16 (AOR: 0.71, 95% CI: 0.65, 0.76) and 2019–21 (AOR: 0.69, 95% CI: 0.64, 0.75). Our study findings revealed social disparities based on caste in odds of receiving full immunisation (ST AOR: 0.84, 95% CI: 0.77, 0.92) in 2015–16. Additionally, a gradual increase in the likelihood of receiving full immunisation was observed with an increase in wealth quintiles when compared to the poorest wealth quintile in 2015–16 (Richest AOR: 1.76, 95% CI: 1.57, 1.98).

**TABLE 2:** Multilevel regression analysis depicting the predictors of full vaccination among 12–23‐month‐old children.

Variables	NFHS‐4		NFHS‐5	
Individual‐level factors	AOR (95% CI)	*p* value	AOR (95% CI)	*p* value
**Maternal age** (in years)				
<25	1		1	
25–34	1.17 (1.09, 1.22)	<0.001	1.13 (1.06, 1.21)	<0.001
≥35	1.16 (1.05, 1.28)	0.003	1.18 (1.05, 1.32)	<0.001
**Maternal education**				
Illiterate	1		1	
Primary	1.30 (1.20,1.40)	<0.001	1.22 (1.11, 1.34)	<0.001
Secondary	1.36 (1.27,1.45)	<0.001	1.36 (1.26, 1.48)	<0.001
Higher	1.42 (1.28,1.58)	<0.001	1.18 (1.05, 1.31)	<0.001
**Wealth index**				
Poorest	1		1	
Poorer	1.23 (1.15, 1.33)	<0.001	1.04 (0.96, 1.12)	0.20
Middle	1.40 (1.29, 1.53)	<0.001	1.15 (1.05, 1.26)	0.003
Richer	1.54 (1.40, 1.70)	<0.001	1.14 (1.02, 1.26)	0.014
Richest	1.76 (1.57, 1.98)	<0.001	1.01 (0.89, 1.14)	0.84
**ANC visits**				
<4	1		1	
≥4	1.58 (1.50,1.67)	<0.001	1.59 (1.50, 1.68)	<0.001
**Place of delivery**				
Home	1		1	
Institutional	1.47 (1.39, 1.57)	<0.001	1.57 (1.45, 1.71)	<0.001
**Pregnancy registration**				
No	1		1	
Yes	2.70 (2.52, 2.90)	<0.001	1.26 (1.18, 1.35)	<0.001
**Parity**				
1 Child	1		1	
More than 1 child	0.84 (0.80, 0.89)	<0.001	0.86 (0.81, 0.92)	<0.001
**Maternal exposure to mass media**				
No	1		1	
Yes	1.18 (1.11, 1.26)	<0.001	1.25 (1.17, 1.35)	<0.001
**Community level**				
**Place of residence**				
Urban	1		—	
Rural	1.12 (1.05, 1.21)	<0.001		
**Religion**				
Hindu	1		1	
Muslim	0.71 (0.65, 0.76)	<0.001	0.69 (0.64, 0.75)	<0.001
Others	1.23 (1.11, 1.36)	<0.001	0.92 (0.82, 1.02)	0.13
**Caste**				
General	1		—	
SC	0.99 (0.92, 1.08)	0.91		
ST	0.84 (0.77, 0.92)	<0.001		
OBC	1.01 (0.94, 1.08)	0.75		
**Region**				
North	1		1	
Central	0.80 (0.74, 0.87)	<0.001	0.75 (0.68, 0.82)	<0.001
East	1.88 (1.72, 2.06)	<0.001	1.18 (1.06, 1.32)	0.003
Northeast	0.60 (0.54, 0.67)	<0.001	0.52 (0.46, 0.57)	<0.001
West	0.56 (0.50, 0.63)	<0.001	0.71 (0.63, 0.80)	<0.001
South	0.84 (0.75, 0.93)	0.001	0.98 (0.87, 1.10)	0.59
**Random effects parameters**				
Cluster effects (95% CI)	0.90 (0.80, 1.01)		0.85 (0.74, 0.97)	
ICC (95% CI)	0.21 (0.19, 0.23)		0.20 (0.18, 0.22)	
LR test *p* value^a^		<0.001		<0.001

Abbreviations: ANC, antenatal care; AOR, adjusted odds ratio; CI, confidence interval; ICC, intra‐class correlation coefficient; NFHS, National Family Health Survey; OBC, Other Backward class; OR, odds ratio; SC, Scheduled Caste; ST, Scheduled Tribe.

^a^ Likelihood ratio test for comparison of the current model with the single‐level logistic model.

Significant regional disparities were observed in the odds of receiving full immunisation, wherein children from eastern parts of India had higher odds (AOR: 1.18, 95% CI: 1.06, 1.32), whereas children from northeastern states had lower odds of receiving full immunisation (AOR: 0.52, 95% CI: 0.46, 0.57) compared to those living in northern parts of India in 2019–21 (NFHS‐5). On the other hand, children belonging to all regions of India, except the eastern part, when compared to northern states, had lower odds of getting full immunisation in 2015–16 (NFHS‐4). Mothers, who attended ≥4 ANC visits (NFHS‐4: AOR: 1.58, 95% CI: 1.50, 1.67; NFHS‐5: AOR: 1.59, 95% CI: 1.50, 1.68), had a pregnancy registration (NFHS‐4: AOR: 2.70, 95% CI: 2.52, 2.90; NFHS‐5: AOR: 1.26, 95% CI: 1.18, 1.35) and had an institutional delivery (NFHS‐4: AOR: 1.47, 95% CI: 1.39, 1.57; NFHS‐5: AOR: 1.59, 95% CI: 1.46, 1.73) were more likely to avail full immunisation for their children over the years. Additionally, maternal exposure to mass media enhanced the odds of getting full immunisation from 2015–16 (AOR: 1.18, 95% CI: 1.11, 1.26) to 2019–21 (AOR: 1.28, 95% CI: 1.20, 1.37). However, those with more than one child had significantly lower odds of receiving full immunisation over the years compared to those with one child (NFHS‐4: AOR: 0.84, 95% CI: 0.80, 0.89; NFHS‐5: AOR: 0.86, 95% CI: 0.81, 0.92). The ICC is quite large (NFHS‐4: ICC: 0.22; NFHS‐5: 0.21), which indicates substantial evidence of clustering effects. Adjusted regression analysis findings showing the factors associated with full immunisation coverage among children aged 12–23 months, stratified by the place of residence and wealth quintile, are shown in Tables S2 and S3, respectively. The geography‐stratified multilevel regression model revealed that the strong association between a higher wealth index and higher odds of full vaccination, which was present in NFHS‐4, was largely attenuated in NFHS‐5 (Table S2). Further, the wealth‐stratified multilevel regression models also revealed that rural residence had significantly higher odds of full vaccination in NFHS‐5 but not in NFHS‐4 (Table S3).

## 4 Discussion

The present analysis from nationally representative country‐level datasets indicates that full immunisation coverage among children in India aged 12–23 months has substantially increased by 14.27% overall, and a 22.8% relative increase, within a 5‐year period (2016–2021), correlating with the multiple phases of the MI programme. The findings from the NFHS‐4 correlated with the increase in coverage resulting from the initial phases of the MI programme (2015–16), whereas NFHS‐5 (2019–21) captures the increase in coverage from the MIK programme (2017–19).

The study findings highlight the critical role of comprehensive maternal healthcare services in improving childhood health outcomes. Our analysis indicates that mothers who engaged more deeply with the healthcare system, from early pregnancy registration to delivery at a health facility, had a greater chance of ensuring their child was fully immunised. These findings indicate that strengthening ANC services enables enhancement of child vaccination coverage through multiple mechanisms, including enhancing maternal awareness, maternal self‐efficacy for navigation of the health system to access child immunisation services, enhanced trust in the health system and continuum of care in the postnatal period. Previously, analysis from the third and fourth rounds of the NFHS using propensity score matching techniques has demonstrated a significantly higher likelihood of mothers of children receiving ANC children to have been fully vaccinated [16, 23]. However, DHS data from Bangladesh did not find a similar association, although a study from Africa observed significantly higher odds of child vaccination in mothers receiving ANC [24, 25].

In the present study, mothers reporting exposure to mass media were more likely to have fully immunised their children. With the growing penetration of mass media in the country, there is widespread utilisation of radio and television sources for the promotion of Indian government health schemes, including the UIP. Parental awareness of the benefits of vaccines in protecting their children against serious disease, recognising the safety of vaccines, and the knowledge of access and availability of free of cost vaccination services enables the development of vaccine confidence and reduces vaccine hesitancy in communities. Moreover, mass media exposure has been significantly associated with increased ANC utilisation by mothers, which can positively impact paediatric vaccination rates [26]. A community‐driven mass media campaign in Malawi also observed improved utilisation of antenatal and postnatal care services [27]. Furthermore, access to mass media, such as radio and TV, has been associated with higher vaccine uptake, as evident from Ethiopia and Nigeria; [28, 29]). Mass media exposure has been linked to significant improvement in maternal health utilisation in multiple South Asian countries, including Bangladesh, Nepal and Pakistan [30, 31].

A major finding of the present study is the absence of significantly differential vaccination coverage in rural compared to urban areas in NFHS‐5, unlike NFHS‐4, where rural areas had significantly lower coverage. In most of South Asia and sub‐Saharan Africa, urban areas tend to have higher antenatal utilisation, paediatric vaccination coverage and less delayed vaccination compared to rural areas [32, 33, 34]. The evidence from NFHS‐5 suggests the MIK has addressed major inequalities and reduced disparities in vaccination coverage between rural and urban parts of India [8, 19]. However, our analysis shows that in some states of the country, vaccine coverage has slightly reduced compared to the previous round of NFHS‐4, which indicates the need for more district‐specific and high‐risk focus across states to achieve the target of 90% and higher child vaccination coverage through the UIP. Recently, a comprehensive district‐level analysis of NFHS‐5 revealed high national vaccination coverage in India masks geographic clustering of under‐vaccination. The vast majority (67.6%) of variation for zero‐dose children is explained by these small‐area ‘clusters’, indicating location is a primary determinant of vaccination status [15].

In urban slums, the MI initiative faced challenges, with only 10% awareness of the programme among respondents, though health workers were key information sources [35]. In rural areas, those living in remote areas, such as tribal populations, also experience a higher risk of diminished coverage and hesitancy [36, 37]. In this study, differential rates of immunisation coverage across religion and caste were observed, undermining equity in the distribution and utilisation of this vital public health good, although in 2019–21, the caste disparity in vaccination improved. In contrast, Muslim cultural beliefs possibly continue to contribute to diminished rates of childhood vaccination in the community, suggesting the need for further engagement with religious leaders of the community to improve vaccination uptake [38].

The present analysis shows that measles vaccine coverage with two doses, required to achieve 95% protection, remains moderate, suggestive of likely requirement for additional catch‐up campaigns in the path towards measles elimination. A recent study estimate suggests 11.5% of children in India have not received any measles vaccination, reflecting a significant proportion of zero‐dose children [39].

The present analysis from two rounds of the NFHS has some important public health policy implications. First, routine immunisation coverage has substantially increased in a 5‐year timeframe, mediated by multiple phases of the Mission Indradhanush Programme with a focus on planning and monitoring by targeting underserved areas and increasing full immunisation rates from 50.5% to 69% in target districts [40]. The NFHS‐5 reveals a demographic transition in child vaccine coverage; rural areas now exhibit significantly higher odds of full vaccination within both lowest and highest wealth quintiles compared to urban counterparts, which was not significant in NFHS‐4, indicative of the success of targeted rural outreach and grassroots mobilisation. To sustain this vital momentum, future iterations of IMI must steadfastly maintain robust rural micro‐planning. However, the concerning stagnation and recent vaccination declines observed in expanding urban centres and comparatively affluent states require the deployment of tailored urban interventions. These steps may include introducing flexible evening vaccination hours to accommodate low‐income working parents reliant on daily wages, aggressively combating vaccine hesitancy through targeted vaccine literacy campaigns, and precisely mapping transient urban slum populations. Upgrading service delivery within these vulnerable demographics is essential to achieving true nationwide immunisation equity.

Second, traditional accessibility factors contributing to reduced vaccination coverage, especially related to rural geography and wealth disparities, have been significantly ameliorated. However, the decline in vaccination coverage in historically high‐performing states like Kerala, Goa and Punjab signals a significant threat to immunisation equity. This backsliding strongly suggests that aggregate state‐level data are masking deep‐seated vulnerabilities and last‐mile gaps. It is crucial to identify the specific clusters of incomplete immunisation, which are likely concentrated among populations, such as the urban poor, migrants and remote communities, even within these relatively affluent states. This finding has direct programmatic implications: Initiatives like the IMI (Kavach) programme, which have traditionally focused on ‘high‐focus’ districts in northern India, must be strategically adapted and expanded to target these newly emerging pockets of underperformance. Finally, addressing vaccine hesitancy in communities with historically lower rates of vaccination is the key to achieving full immunisation. As mass media have already shown promise in improving vaccine confidence, as evident from these study findings, the deployment of social media for the same purpose may warrant consideration [41].

### 4.1 Strengths and Limitations

The methodological rigour of this study is a notable strength. By utilising a nationally representative dataset, we ensure the broad applicability of our results. The analysis advances prior work [42] by factoring in both religion and caste as important risk factors for non‐immunisation. Our use of a multilevel hierarchical model is particularly significant; it not only accounts for data clustering but also allows us to provide more nuanced estimates by examining the interplay between individual‐ and community‐level variables. This integrated approach, which grounds our statistical model in a standardised theoretical framework, ensures that our findings are not only comprehensive but also conceptually robust. Further, this analysis established the elimination of rural residence as a significant factor for lower child vaccination odds in NFHS‐5, a contrast to NFHS‐4, which suggests that national health policies have become more effective at reducing geographical disparities and therefore more equitable. This testified to a notable achievement in ensuring equitable access to healthcare.

The limitation of the study is that the NFHS schedule did not include any specific questions on reasons for vaccine hesitancy among the unimmunised. Moreover, information on rotavirus and pneumococcal vaccination status was unavailable, as they were introduced more recently in the NIS. The cross‐sectional design fails to provide any causal association.

## 5 Conclusion

Full immunisation coverage among children aged 12–23 months in India increased from 62.5% in 2015–16 to 76.8% in 2019–21. Key predictors of full immunisation included higher maternal education and age, greater household wealth, institutional delivery, ANC (≥4 visits), pregnancy registration and media exposure, whereas lower odds were associated with being Muslim and having multiple children. Although improvements were observed in historically underperforming states like Uttar Pradesh and Bihar, regional disparities remain across southern, northern and northeastern states. Targeted strategies are needed to address persistent gaps among specific groups, including religious minorities and multi‐child households. Future research should explore context‐specific sociocultural barriers and evaluate the effectiveness of tailored community‐based interventions in improving vaccine uptake in underserved regions.

## Author Contributions

**Satyajit Kundu**: investigation, validation, visualisation, writing – review and editing, methodology. **Mansi Malik**: conceptualisation, methodology, data curation, formal analysis, writing – original draft, software. **Siaa Girotra**: validation, data curation, visualisation, writing – original draft. **Saurav Basu**: conceptualisation, methodology, investigation, validation, writing – original draft, writing – review and editing, supervision, software.

## Funding

The authors have nothing to report.

## Ethics Statement

The authors assert that all procedures contributing to this work comply with the ethical standards of the relevant national and institutional committees on human experimentation and with the Helsinki Declaration of 1975, as revised in 2008.

## Conflicts of Interest

The authors declare no conflicts of interest.

## Supporting information




**Supporting info**: puh270329‐sup‐0001‐SuppMat.docx

## Data Availability

This study analyzed secondary data from the fourth and fifth rounds of the National Family Health Survey (NFHS) in India. These datasets are part of the Demographic and Health Surveys (DHS) Program and are available in the public domain. Access to the datasets can be granted to researchers upon registration and formal request via the DHS website (https://dhsprogram.com).
