# Supplementary material for: Comprehensive Immunisation Among Children Aged 12–23 Months in India—A Multilevel Modelling Analysis From the National Family Health Survey (2015–21)
Source: Public Health Chall. 2026 Aug 11;5(3):e70329. doi: 10.1002/puh2.70329 (PMC13460399; doi:10.1002/puh2.70329)
Supplement: Supplementary file 1 [file PUH2-5-e70329-s001.docx]

**Supplementary File**

**Table S1**. Bivariate association of vaccination coverage (full versus incomplete) by individual and community-level variables

| **Variables** | **NFHS-4** |  | **NFHS-5** |  |
| --- | --- | --- | --- | --- |
|  | **Full immunization** |  | **Full Immunization** |  |
| **Individual-level Factors** | **OR (95% CI)** | **p-value** | **OR (95% CI)** | **p-value** |
| **Sex of Child** |  |  |  |  |
| Male | 1 |  | 1 |  |
| Female | 1.00 (0.95,1.05) | 0.97 | 0.95 (0.89, 1.01) | 0.09 |
| **Maternal Age** (in years) |  |  |  |  |
| <25 | 1 |  | 1 |  |
| 25-34 | 0.94 (0.89, 0.99) | 0.043 | 0.98 (0.92, 1.05) | 0.64 |
| >=35 | 0.72 (0.63, 0.81) | <0.001 | 0.83 (0.72, 0.95) | 0.006 |
| **Maternal Education** |  |  |  |  |
| No Education | 1 |  | 1 |  |
| Primary | 1.44 (1.32, 1.56) | <0.001 | 1.41 (1.27, 1.57) | <0.001 |
| Secondary | 1.87 (1.75, 2.00) | <0.001 | 1.75 (1.62, 1.89) | <0.001 |
| Higher | 2.27 (2.04, 2.54) | <0.001 | 1.68 (1.50, 1.89) | <0.001 |
| **Wealth Index** |  |  |  |  |
| Poorest | 1 |  | 1 |  |
| Poorer | 1.36 (1.27, 1.46) | <0.001 | 1.25 (1.15, 1.36) | <0.001 |
| Middle | 1.60 (1.48, 1.74) | <0.001 | 1.59 (1.45, 1.75) | <0.001 |
| Richer | 1.80 (1.63, 1.97) | <0.001 | 1.57 (1.41, 1.75) | <0.001 |
| Richest | 2.08 (1.86, 2.31) | <0.001 | 1.50 (1.35, 1.67) | <0.001 |
| **ANC Visits** |  |  |  |  |
| <4 | 1 |  | 1 |  |
| >=4 | 2.01 (1.90, 2.14) | <0.001 | 1.73 (1.61, 1.85) | <0.001 |
| **Place of Delivery** |  |  |  |  |
| Home | 1 |  | 1 |  |
| Institutional | 2.04 (1.90, 2.19) | <0.001 | 1.92 (1.75, 2.11) | <0.001 |
| **Pregnancy registration** |  |  |  |  |
| No | 1 |  | 1 |  |
| Yes | 2.93 (2.70, 13.17) | <0.001 | 1.65 (1.52, 1.78) | <0.001 |
| **Parity** |  |  |  |  |
| One child | 1 |  | 1 |  |
| More than one child | 0.69 (0.65, 0.73) | <0.001 | 0.77 (0.71, 0.82) | <0.001 |
| **Exposure to mass media** |  |  |  |  |
| No | 1 |  | 1 |  |
| Yes | 1.74 (1.64, 1.84) | <0.001 | 1.57 (1.47, 1.68) | <0.001 |
| **Community-Level Factors** |  |  |  |  |
| **Place of Residence** |  |  |  |  |
| Urban | 1 |  | 1 |  |
| Rural | 0.89 (0.82, 0.96) | 0.005 | 1.08 (0.99, 1.18) | 0.08 |
| **Religion** |  |  |  |  |
| Hindu | 1 |  | 1 |  |
| Muslim | 0.72 (0.67, 0.79) | <0.001 | 0.71 (0.64, 0.78) | <0.001 |
| Others | 1.32 (1.14, 1.54) | <0.001 | 1.12 (0.96, 1.30) | 0.13 |
| **Caste** |  |  |  |  |
| General | 1 |  | 1 |  |
| SC | 0.98 (0.89, 1.08) | 0.74 | 1.04 (0.93, 1.15) | 0.48 |
| ST | 0.73 (0.66, 0.81) | <0.001 | 1.02 (0.89, 1.17) | 0.78 |
| OBC | 0.93 (0.85, 1.00) | 0.07 | 1.05 (0.96, 1.16) | 0.26 |
| **Region** |  |  |  |  |
| North | 1 |  | 1 |  |
| Central | 0.65 (0.60, 0.71) | <0.001 | 0.65 (0.59, 0.72) | <0.001 |
| East | 1.32 (1.20, 1.46) | <0.001 | 0.92 (0.83, 1.02) | 0.12 |
| Northeast | 0.57 (0.50, 0.63) | <0.001 | 0.51 (0.46, 0.58) | <0.001 |
| West | 0.68 (0.60, 0.77) | <0.001 | 0.75 (0.64, 0.87) | <0.001 |
| South | 1.22 (1.09, 1.37) | 0.001 | 1.17 (1.03, 1.32) | 0.01 |

CI: Confidence interval; OBC: Other backward class; OR: Odds Ratio; SC: Scheduled caste; ST: Scheduled tribe

**Table S2.** Adjusted Multilevel analysis stratified by residence

|  | **NFHS 4** | | | | **NFHS 5** | | | |
| --- | --- | --- | --- | --- | --- | --- | --- | --- |
| **Variables** | **Urban** | | **Rural** | | **Urban** | | **Rural** | |
|  | aOR (95% CI) | p-value | aOR (95% CI) | p-value | aOR (95% CI) | p-value | aOR (95% CI) | p-value |
| **Individual-level Factors** |  |  |  |  |  |  |  |  |
| **Sex of child** |  |  |  |  |  |  |  |  |
| Male | - |  | 1 |  | 1 |  | 1 |  |
| Female |  |  | 1.02 (0.97,1.08) | 0.408 | 1.08 (0.96, 1.21) | 0.22 | 0.93 (0.87, 0.98) | 0.02 |
| **Maternal Age** (in years) |  |  |  |  |  |  |  |  |
| <25 | - |  | 1 |  | - |  | 1 |  |
| 25-34 |  |  | 1.17 (1.10,1.36) | <0.001 |  |  | 1.15 (1.07, 1.24) | <0.001 |
| >=35 |  |  | 1.18 (1.05,1.31)) | <0.001 |  |  | 1.20 (1.06, 1.37) | 0.005 |
| **Maternal Education** |  |  |  |  |  |  |  |  |
| Illiterate | 1 |  | 1 |  | 1 |  | 1 |  |
| Primary | 1.43 (1.19,1.71) | <0.001 | 1.25 (1.15,1.36) | <0.001 | 1.16 (0.89, 1.51) | 0.28 | 1.23 (1.11, 1.36) | <0.001 |
| Secondary | 1.43 (1.23,1.66) | <0.001 | 1.36 (1.26,1.46) | <0.001 | 1.40 (1.14, 1.73) | 0.001 | 1.37 (1.26, 1.50) | <0.001 |
| Higher | 1.56 (1.29,1.89) | <0.001 | 1.43 (1.26,1.63) | <0.001 | 1.21 (0.95, 1.54) | 0.12 | 1.25 (1.09, 1.43) | 0.001 |
| **Wealth Index** |  |  |  |  |  |  |  |  |
| Poorest | 1 |  | 1 |  | 1 |  | 1 |  |
| Poorer | 1.22 (0.95,1.59) | 0.122 | 1.26 (1.17,1.36) | <0.001 | 1.22 (0.89, 1.67) | 0.21 | 1.06 (0.97, 1.15) | 0.17 |
| Middle | 1.18 (0.92,1.51) | 0.193 | 1.50 (1.37,1.64) | <0.001 | 1.54 (1.14, 2.09) | 0.006 | 1.16 (1.10, 1.28) | 0.003 |
| Richer | 1.30 (1.01,1.67) | 0.039 | 1.66 (1.49,1.85) | <0.001 | 1.75 (1.28, 2.37) | <0.001 | 1.13 (1.01, 1.27) | 0.04 |
| Richest | 1.54 (1.18,2.01) | <0.001 | 1.78 (1.55,2.04) | <0.001 | 1.66 (1.20, 2.27) | 0.002 | 1.05 (0.90, 1.23) | 0.51 |
| **ANC Visits** |  |  |  |  |  |  |  |  |
| <4 | 1 |  | 1 |  | 1 |  | 1 |  |
| >=4 | 1.64 (1.47,1.82) | <0.001 | 1.60 (1.50,1.70) | <0.001 | 1.35 (1.18, 1.54) | <0.001 | 1.66 (1.56, 1.77) | <0.001 |
| **Place of Delivery** |  |  |  |  |  |  |  |  |
| Home | 1 |  | 1 |  | 1 |  | 1 |  |
| Institutional | 1.45 (1.24,1.70) | <0.001 | 1.48 (1.39,1.59) | <0.001 | 1.61 (1.25, 2.07) | <0.001 | 1.59 (1.46, 1.74) | <0.001 |
| **Pregnancy registration** |  |  |  |  |  |  |  |  |
| No | 1 |  | 1 |  | 1 |  | 1 |  |
| Yes | 1.96 (1.71,2.25) | <0.001 | 2.92 (2.69,3.16) | <0.001 | 1.38 (1.14, 1.66) | 0.001 | 1.25 (1.16, 1.35) | <0.001 |
| **Parity** |  |  |  |  |  |  |  |  |
| 1 | 1 |  | 1 |  | 1 |  | 1 |  |
| >2 | 0.81 (0.73,0.89) | <0.001 | 0.86 (0.81,0.92) | <0.001 | 0.85 (0.75, 0.96) | 0.009 | 0.87 (0.81, 0.94) | <0.001 |
| **Maternal exposure to mass media** |  |  |  |  |  |  |  |  |
| No | 1 |  | 1 |  | 1 |  | 1 |  |
| Yes | 1.16 (0.96,1.39) | 0.111 | 1.18 (1.10,1.26) | <0.001 | 1.46 (1.21, 1.76) | <0.001 | 1.27 (1.18, 1.36) | <0.001 |
| **Community Level Factors** |  |  |  |  |  |  |  |  |
| **Region** |  |  |  |  |  |  |  |  |
| North | 1 |  | 1 |  | 1 |  | 1 |  |
| Central | 0.76 (0.65,0.88) | 0.001 | 0.81 (0.74,0.89) | <0.001 | 0.77 (0.63, 0.94) | 0.009 | 0.75 (0.67, 0.84) | <0.001 |
| East | 1.24 (1.02,1.51) | 0.026 | 2.04 (1.84,2.26) | <0.001 | 1.07 (0.84, 1.37) | 0.57 | 1.22 (1.08, 1.37) | 0.001 |
| Northeast | 0.67 (0.55,0.81) | <0.001 | 0.59 (0.53,0.66) | <0.001 | 0.60 (0.59, 0.92) | <0.001 | 0.51 (0.45, 0.58) | <0.001 |
| West | 0.48 (0.39,0.59) | <0.001 | 0.55 (0.48,0.63) | <0.001 | 0.73 (0.58, 0.92) | 0.007 | 0.76 (0.66, 0.87) | <0.001 |
| South | 0.89 (0.75,1.07) | 0.247 | 0.77 (0.68,0.87) | <0.001 | 0.95 (0.78, 1.17) | 0.66 | 1.04 (0.91, 1.20) | 0.51 |
| **Random Effects parameters** |  |  |  |  |  |  |  |  |
| Cluster effects (95% CI) | 0.89 (0.71,1.12) |  | 0.89 (0.79,1.00) |  | 0.98 (0.73, 1.33) |  | 0.82 (0.70, 0.95) |  |
| ICC (95% CI) | 0.21 (0.17,0.25) |  | 0.21 (0.19,0.23) |  | 0.23 (0.18, 0.29) |  | 0.19 (0.17, 0.22) |  |
| LR test p-value* | <0.001 |  | <0.001 |  |  | <0.001 |  | <0.001 |

AIC: Akaike information criterion; ANC: Antenatal care; aOR: adjusted Odds ratio; BIC: Bayesian information criterion; CI: Confidence interval; ICC: Intraclass correlation coefficient

*Likelihood ratio test for comparison of the current model with the single level logistic model

**Table S3.** Adjusted Multilevel analysis stratified by SES

|  | **NFHS 4** | | | | **NFHS 5** | | | |
| --- | --- | --- | --- | --- | --- | --- | --- | --- |
| **Variables** | **Wealth quintile 1** | | **Wealth quintile 5** | | **Wealth quintile 1** | | **Wealth quintile 5** | |
|  | OR (95% CI) | p-value | OR (95% CI) | p-value | OR (95% CI) | p-value | OR (95% CI) | p-value |
| **Individual-level Factors** |  |  |  |  |  |  |  |  |
| **Sex of child** | - |  | - |  |  |  | - |  |
| Male |  |  |  |  | 1 |  |  |  |
| Female |  |  |  |  | 0.89 (0.81, 0.99) | 0.03 |  |  |
| **Maternal Age** (in years) |  |  |  |  |  |  |  |  |
| <25 | 1 |  | - |  | - |  | 1 |  |
| 25-34 | 1.19 (1.06, 1.33) | 0.002 |  |  |  |  | 1.05 (0.87, 1.27) | 0.57 |
| >=35 | 1.10 (0.93, 1.31) | 0.25 |  |  |  |  | 0.92 (0.67, 1.27) | 0.63 |
| **Maternal Education** |  |  |  |  |  |  |  |  |
| Illiterate | 1 |  | - |  | 1 |  |  |  |
| Primary | 1.46 (1.29, 1.66) | <0.001 |  |  | 1.16 (1.01, 1.35) | 0.04 |  |  |
| Secondary | 1.44 (1.27, 1.62) | <0.001 |  |  | 1.26 (1.10, 1.42) | <0.001 |  |  |
| Higher | 2.12 (1.23, 3.63) | 0.006 |  |  | 1.18 (0.76, 1.84) | 0.45 |  |  |
| **Residence** |  |  |  |  |  |  |  |  |
| Urban | - |  | 1 |  | 1 |  | 1 |  |
| Rural |  |  | 1.08 (0.94, 1.26) | 0.26 | 1.72 (1.30, 2.27) | <0.001 | 1.23 (1.04, 1.46) | 0.01 |
| **ANC Visits** |  |  |  |  |  |  |  |  |
| <4 | 1 |  | 1 |  | 1 |  |  |  |
| >=4 | 1.62 (1.45, 1.82) | <0.001 | 1.78 (1.52, 2.08) | <0.001 | 1.92 (1.72, 2.16) | <0.001 |  |  |
| **Place of Delivery** |  |  |  |  |  |  |  |  |
| Home | 1 |  | 1 |  | 1 |  | 1 |  |
| Institutional | 1.47 (1.33, 1.62) | <0.001 | 1.71 (1.25, 2.34) | 0.001 | 1.62 (1.43, 1.83) | <0.001 | 2.16 (1.36, 3.46) | <0.001 |
| **Pregnancy registration** |  |  |  |  |  |  |  |  |
| No | 1 |  | 1 |  | 1 |  |  |  |
| Yes | 3.25 (2.87, 3.67) | <0.001 | 2.09 (1.68, 2.59) | <0.001 | 1.29 (1.15, 1.45) | <0.001 |  |  |
| **Parity** |  |  |  |  |  |  |  |  |
| 1 | 1 |  | 1 |  | 1 |  | 1 |  |
| >2 | 0.94 (0.83, 1.06) | <0.34 | 0.78 (0.68, 0.89) | <0.001 | 0.99 (0.87, 1.12) | 0.85 | 0.76 (0.64, 0.90) | 0.001 |
| **Maternal exposure to mass media** |  |  |  |  |  |  |  |  |
| No | 1 |  | - |  | 1 |  | - |  |
| Yes | 1.10 (0.99, 1.21) | <0.06 |  |  | 1.22 (1.09, 1.36) | <0.001 |  |  |
| **Community Level Factors** |  |  |  |  |  |  |  |  |
| **Caste** |  |  |  |  |  |  |  |  |
| General | 1 |  | - |  | 1 |  | - |  |
| SC | 1.37 (1.13, 1.66) | 0.001 |  |  | 1.07 (0.85, 1.35) | 0.53 |  |  |
| ST | 1.27 (1.05, 1.53) | 0.011 |  |  | 1.31 (1.05, 1.64) | 0.02 |  |  |
| OBC | 1.40 (1.17, 1.69) | <0.001 |  |  | 1.01 (0.81, 1.26) | 0.89 |  |  |
| **Region** |  |  |  |  |  |  |  |  |
| North | 1 |  | 1 |  | 1 |  | 1 |  |
| Central | 1.03 (0.85, 1.25) | 0.74 | 0.65 (0.53, 0.78) | <0.001 | 0.80 (0.64, 1.02) | 0.08 | 0.63 (0.51, 0.78) | <0.001 |
| East | 2.45 (2.02, 2.97) | <0.001 | 0.82 (0.60, 1.13) | 0.23 | 1.33 (1.05, 1.69) | 0.02 | 0.78 (0.54, 1.13) | 0.18 |
| Northeast | 0.70 (0.55, 0.89) | 0.003 | 0.50 (0.38, 0.66) | <0.001 | 0.54 (0.41, 0.70) | <0.001 | 0.47 (0.32, 0.71) | <0.001 |
| West | 0.64 (0.47, 0.87) | 0.004 | 0.46 (0.35, 0.60) | <0.001 | 0.85 (0.61, 1.19) | 0.36 | 0.84 (0.64, 1.10) | 0.22 |
| South | 1.62 (1.11, 2.35) | 0.012 | 0.70 (0.55, 0.88) | 0.002 | 0.90 (0.61, 1.35) | 0.64 | 0.90 (0.71, 1.15) | 0.41 |
| **Random Effects parameters** |  |  |  |  |  |  |  |  |
| Cluster effects (95% CI) | 0.73 (0.56, 0.96) |  | 1.38 (0.94, 2.02) |  | 0.89 (0.67, 1.20) |  | 1.33 (0.85, 2.09) |  |
| ICC (95% CI) | 0.18 (0.15, 0.22) |  | 0.29 (0.22, 0.38) |  | 0.21 (0.17, 0.28) |  | 0.29 (0.20, 0.39) |  |
| LR test p-value* |  | <0.001 |  | <0.001 |  | <0.001 |  | <0.001 |

ANC: Antenatal care; aOR: adjusted Odds ratio; CI: Confidence interval; ICC: Intraclass correlation coefficient; SES: Socioeconomic status

*Likelihood ratio test for comparison of the current model with the single-level logistic model

**Figure S1**. State-wise coverage (%) of BCG vaccine in NFHS-4 and NFHS-5

**Figure S2**. State-wise coverage (%) of Hepatitis B vaccine in NFHS-4 and NFHS-5

**Figure S3**. State-wise coverage (%) of measles vaccine in NFHS-4 and NFHS-5

**Figure S4.** State-wise coverage (%) of OPV (0-3 doses) vaccine in NFHS-4 and NFHS-5

**Figure S5.** State-wise coverage (%) of DPT (1-3 doses) vaccine in NFHS-4 and NFHS-5

**Figure S6**. State-wise change in immunisation coverage (%) in NFHS-4 and NFHS-5

**Figure S7.** Full immunisation coverage among children aged 12-23 months stratified by age and sex, (2019-21)

**Figure S8**. Full immunisation coverage among children aged 12-23 months stratified by age and sex, (2015-16)
